# Structural barriers drive the intention-action gap in hepatitis B prevention among migrants in Shandong Province, China: a triangulated study of serological, spatial, and behavioral data

**DOI:** 10.1186/s12889-026-28627-z

**Published:** 2026-07-18

**Authors:** Yuan Sun, Bingyang Li, Na Li, Feng Hao, Peng Chen

**Affiliations:** 1 Department of Public Health, Shandong College of Traditional Chinese Medicine, Yantai, Shandong 264100 China; 2 Department of Immunization Prevention, Yantai Center for Disease Control and Prevention, Yantai, Shandong 264000 China

**Keywords:** Hepatitis B, Internal migrant workers, Intention-action gap, Structural barriers, Structural Equation Modeling, Spatial analysis, Care cascade

## Abstract

**Background:**

Hepatitis B virus (HBV) infection remains a major public health challenge in China. Migrant workers constitute a highly vulnerable population due to high geographic mobility and occupational vulnerability. Despite public health education efforts, a notable intention-action gap disrupts the HBV care cascade. This study integrates serological, spatial, and behavioral data to investigate whether this gap is primarily driven by cognitive deficits or interrupted by underlying structural barriers.

**Methods:**

A triangulated cross-sectional study was conducted in Yantai, China, involving structured questionnaire surveys (N = 1,184) and serological testing (N = 2,364). The spatial distribution of regional anti-HBs positive rates was mapped using Geographic Information Systems (GIS). An adapted Health Belief Model was tested using Structural Equation Modeling (SEM) with a Robust Maximum Likelihood estimator to evaluate the disruptive effects of objective structural constraints—specifically, time poverty, economic constraints, and systemic lack of medical insurance.

**Results:**

The serologically confirmed anti-HBs positive rate was 51.10% (1,208/2,364), substantially lower than the 70% threshold recommended by the World Health Organization. Spatial analysis revealed significant geographic heterogeneity (χ² = 74.38, P < 0.001), with higher immunity concentrated in large-scale industrial belts (67.33%) and lower immunity clustered in dispersed service-sector districts (36.81%). SEM results indicated that while external cues strongly predicted HBV knowledge (β = 0.894), knowledge had only a marginal effect on preventive intention (β = 0.192). Preventive intention significantly predicted actual behavior (β = 0.655). However, this motivational process was severely undermined by structural barriers, predominantly time poverty (β = -0.563, P < 0.001), followed by economic constraints (β = -0.278, P < 0.001) and lack of medical insurance (β = -0.056, P = 0.030).

**Conclusions:**

The intention-action gap in HBV prevention among migrant workers is primarily driven by socioeconomic structural barriers—most notably time poverty—while basic HBV knowledge remains a foundational but insufficient condition for preventive action. Achieving the goal of viral hepatitis elimination requires a shift from individual cognitive persuasion to structural interventions, emphasizing workplace-based service delivery, spatially targeted resource allocation, and insurance reforms to alleviate out-of-pocket and opportunity costs.

**Supplementary Information:**

The online version contains supplementary material available at https://doi.org/10.1186/s12889-026-28627-z.

## Background

Hepatitis B virus (HBV) infection remains a pressing global public health challenge. China currently bears the world’s heaviest burden of HBV, accounting for approximately one-third of all global chronic infections [1, 2]. Although historic progress has been made through universal neonatal vaccination programs, existing adult infections and horizontal transmission continue to pose substantial public health concerns. Notably, rapid urbanization in China has generated a massive population of internal migrant workers. Characterized by high geographic mobility, heavy occupational burdens, and systemic barriers to medical insurance coverage, this demographic constitutes a highly vulnerable link in the HBV prevention network [3].

Achieving the World Health Organization’s (WHO) 2030 goal of eliminating viral hepatitis depends fundamentally on the integration of the “prevention, screening, diagnosis, and treatment” care cascade [4–6]. Current public health interventions targeting high-risk populations frequently utilize sociopsychological frameworks, such as the Health Belief Model (HBM) [7], to enhance behavioral motivation through external cues to action. However, public health practice reveals a critical discrepancy: individuals’ high self-reported willingness to undergo screening and vaccination often fails to translate into actual clinical protective behaviors. This notable “intention-action gap” forms a major bottleneck disrupting the HBV care cascade [8]. Yet, whether this discrepancy is primarily driven by cognitive dimensions or interrupted by underlying structural barriers—such as severe time constraints, economic limitations, and lack of medical insurance—remains insufficiently quantified in current literature [9, 10].

Previous studies investigating the willingness of migrant populations to engage in HBV prevention exhibit distinct methodological limitations. First, the majority of research relies solely on cross-sectional, self-reported survey data, which is highly susceptible to social desirability and recall biases, often leading to an overestimation of true protection levels [11]. Second, such sociological surveys typically lack objective clinical laboratory evidence (e.g., serological immune markers) for baseline validation. Finally, behavioral studies rarely integrate micro-level psychological models with macro-level geographic information systems (GIS), thereby overlooking spatial disparities in immune protection and infection risks caused by the uneven distribution of healthcare resources.

To address these research gaps, the present study adopts a triangulation design that integrates behavioral survey data, serological biomarkers, and spatial analysis [12], focusing on the migrant worker population in Yantai City, China. The specific objectives are to: (1) construct a robust Structural Equation Model (SEM) to quantitatively test the intention-action gap and evaluate the disruptive effects of objective structural barriers; (2) incorporate real-world seroepidemiological data to cross-validate self-reported behaviors and establish the true baseline immunity ; and (3) utilize spatial heatmaps to visually map the geographic disparities in regional immunization rates. By synthesizing multi-source heterogeneous data, this study aims to look beyond superficial willingness and reveal the systemic barriers impeding the care cascade, thereby providing high-level evidence for precise public health policymaking.

## Methods

### Study design and setting

A cross-sectional, triangulated study integrating questionnaire surveys and serological testing was conducted between January and March 2025 in Yantai City, Shandong Province, China. To comprehensively assess both the subjective psychosocial determinants and the objective biological status of HBV prevention, data were collected through two sequential cross-sectional survey components targeting the same source population. Before field implementation, the research team communicated with enterprise and worksite managers to obtain organizational support. The online questionnaire survey was conducted first: questionnaire links were distributed by managers to migrant workers. After completion of the questionnaire survey, the research team visited the corresponding enterprises and worksites to conduct on-site venous blood collection and HBV serological testing.

The inclusion criteria for all participants were: (1) age 20 or older; (2) not a registered resident of Yantai; (3) currently employed in a non-agricultural sector in Yantai (e.g., manufacturing plants, construction sites). All participants signed an informed consent form prior to enrollment, and the study protocol was reviewed and approved by the Preventive Medicine Ethics Committee of the Yantai Center for Disease Control and Prevention (No. YYLLS 2024-23).

### Participants and sampling

Questionnaire Survey: A cross-sectional survey design was adopted. In each district of Yantai City, two locations with high concentrations of migrant workers (including factories, mines, and construction sites) were selected, and participants were recruited using convenience sampling. The survey was conducted via an online questionnaire (on the Wenjuanxing platform), with the questionnaire link being sent directly by enterprise and site managers to workers who met the inclusion criteria. The questionnaire was designed to be completed anonymously, with each IP address limited to one response. Prior to the formal investigation, a pilot study involving 50 migrant workers was conducted to refine the questionnaire phrasing. The required minimum sample size was calculated as 335, based on an estimated HBV knowledge rate of 68% (*P* = 0.68) derived from previous literature [13], a 95% confidence interval (Z = 1.96), and a margin of error of 0.05 (d = 0.05). Considering potential non-responses, the target recruitment was set to approximately 1,000 individuals.

Serological Testing: Following the questionnaire survey, on-site venous blood collection was conducted at the corresponding migrant worker sites. A trained nurse collected 5 mL of venous blood; after serum separation, the five hepatitis B markers (HBsAg, anti-HBs, HBeAg, anti-HBe, and anti-HBc) were tested using chemiluminescence (instrument: Abbott Alinity i; reagent: Abbott Hepatitis B Virus Test Kit). Result interpretation strictly follows relevant guidelines and the kit instructions: (1) Anti-HBs positivity is defined as a quantitative result ≥ 10 mIU/mL, indicating immune protection; (2) HBsAg positivity is defined as a quantitative result ≥ 0.05 IU/mL, suggesting active infection. The minimum sample size for serology was calculated as 1,067, assuming an anti-HBs positive rate of 50% (*P* = 0.50) [14], with a margin of error of 0.03. Supported by a dedicated funding grant, free HBV five-panel testing was offered, which highly motivated participation. Consequently, 2,364 workers were successfully recruited, substantially exceeding the minimum requirement.

The two survey components were conducted sequentially within the same selected enterprise/worksite populations. This design was adopted to combine anonymous self-reported behavioral data with objective serological evidence while protecting participant privacy. Because the questionnaire was anonymous and no shared identifier was retained between questionnaire and serological testing records, individual-level linkage could not be established and the exact overlap proportion could not be calculated. Nevertheless, both samples were drawn from the same selected enterprise/worksite populations and were broadly comparable in age and place of household registration (Table 1).

**Table 1:** Basic characteristics of participants

Characteristic	Questionnaire Survey (*N* = 1184)	Serological Testing (*N* = 2364)	*P*-value
Sex, n (%)			
Male	657(55.49)	1394(58.97)	0.047
Female	527(44.51)	970(41.03)	
Age, n (%)			
20–25 years	164(13.85)	300(12.69)	0.085
26–35 years	266(22.47)	580(24.53)	
36–45 years	325(27.45)	660(27.92)	
46–55 years	245(20.69)	470(19.88)	
> 55 years	184(15.54)	354(14.98)	
Region, n (%)			
Same province	506(42.74)	1050(44.42)	0.099
Other provinces	678(57.26)	1314(55.58)	
Industry, n (%)			
Manufacturing	678(57.26)	-	-
Construction	128(10.81)	-	
Service	160(13.51)	-	
Transportation	18(1.53)	-	
Other	200(16.89)	-	
Monthly income, n (%)			
< 3000 RMB	115(9.71)	-	-
3000–5000 RMB	631(53.30)	-	
5001–10,000 RMB	346(29.22)	-	
> 10,000 RMB	92(7.77)	-	
Medical insurance, n (%)			
No	1066(90.03)	-	-
Yes	118(9.97)	-	

### Data collection and measurements

A structured questionnaire developed specifically for this study (provided as Supplementary Material S1) was used to collect the following information:

Demographic characteristics: gender, age, place of household registration (hukou), current industry sector, and monthly average income, and whether having local basic medical insurance in Yantai.

HBV‑related knowledge and behavior history: awareness of hepatitis B, sources of information (television/radio, internet, community outreach, family/friends), knowledge about transmission routes and preventive measures, and self‑reported history of HBV screening and vaccination.

Preventive intentions: willingness to undergo HBV screening; to receive vaccination if tested uninfected and without antibodies; to undergo diagnostic confirmation if screening suggested possible infection; and to receive treatment if diagnosed with hepatitis B. Items were rated on a four‑point Likert scale (1 = very unwilling, 4 = very willing). The scales demonstrated good internal consistency, with a Cronbach’s α of 0.85 for the behavioral intention dimension.

Barriers to the care cascade: For participants who responded “unwilling” to any service, a multiple-choice question assessed the primary reasons, encompassing both psychosocial factors (e.g., fear of discrimination, perceived unnecessity) and objective structural constraints. In the operationalization of structural barriers for subsequent modeling, only socioeconomic and systemic impediments (i.e., time poverty, inability to afford costs, and lack of medical insurance) were included.

### Structural Equation Modeling (SEM) variables

To evaluate the “intention-action gap” and the disruptive effects of structural barriers, latent variables and exogenous covariates were operationalized within an adapted Health Belief Model (HBM) framework. Due to severe floor effects (positive response rates < 1.2%), subjective fear indicators—including fear of testing, discrimination, and job loss—were excluded from the measurement model. Given the predominantly asymptomatic nature of chronic HBV infection, the traditional “Perceived Threat” construct was replaced by objective “HBV Knowledge” to serve as a stable proxy for cognitive appraisal.

The finalized latent constructs consisted of:

- 1. Cues to Action, measured by four binary items regarding information sources;
- 2. HBV Knowledge, comprising two indicators assessing transmission routes and preventive measures (scored 0–4);
- 3. Preventive Intention, evaluated via four Likert-type items reflecting willingness to screen, vaccinate, undergo diagnosis, and receive treatment across the care cascade;
- 4. Actual Behavior, defined by self-reported history of HBV screening and vaccination.

Three objective structural barriers—time poverty, economic constraints, and lack of medical insurance—were modeled as independent, exogenous observed covariates predicting preventive intention. Time poverty was defined as the inability to take time off work; economic constraints as the inability to afford out-of-pocket costs; and lack of insurance as having no localized basic medical insurance in Yantai. To account for the questionnaire’s skip logic, participants who expressed willingness to utilize services were assigned a value of “0” for these barriers for modeling purposes. The hypothesized pathways were grounded in the adapted HBM [7] and Knowledge-Attitude-Practice models [15]. Alternative pathways, such as direct effects of barriers on actual behavior, were excluded from model estimation because barrier data were elicited via skip-logic solely from participants expressing preventive unwillingness.

### Statistical analysis

Descriptive statistics and Chi-square tests were conducted to summarize demographic characteristics and compare serological positive rates across groups using SPSS version 20.0 (IBM Corp., Armonk, NY, USA). Spatial distribution mapping of the regional anti-HBs positive rates was generated using the sf, ggplot2, and viridis packages in R software (version 4.3.2).

Structural Equation Modeling (SEM) was employed to test the adapted HBM framework and evaluate the effects of structural barriers. Prior to model estimation, items measuring behavioral intention were reverse-coded so that higher scores consistently reflected stronger preventive motivation. To accommodate the categorical nature of the survey items and correct for potential non-normality, the model was estimated using the Robust Maximum Likelihood (MLR) estimator [16].

Before testing the structural relationships, a Confirmatory Factor Analysis (CFA) was conducted to verify the reliability and construct validity of the measurement model. Internal consistency and construct reliability were evaluated using Cronbach’s α and Composite Reliability (CR), with values ≥ 0.70 indicating acceptable reliability. Convergent validity was assessed using the Average Variance Extracted (AVE), with a recommended threshold of ≥ 0.50.

Subsequently, the structural model was evaluated. Guided by Modification Indices (MI) and clinical rationale, a residual covariance was specified between diagnostic and treatment intentions to account for their shared variance within the post-screening care pathway. Model fit was assessed using robust fit indices, with acceptable fit defined as a Comparative Fit Index (CFI) ≥ 0.90, Tucker-Lewis Index (TLI) ≥ 0.90, Root Mean Square Error of Approximation (RMSEA) < 0.08, and Standardized Root Mean Square Residual (SRMR) < 0.08 [17]. All SEM analyses and reliability calculations were performed using the lavaan package (version 0.6–21) and the semTools package in R software (version 4.3.2), with two-tailed statistical significance defined at α = 0.05.

## Results

### Characteristics of the study population

A total of 1,184 valid questionnaires were collected, yielding an effective response rate of 98.58%. As shown in Table 1, the questionnaire respondents were predominantly male (55.49%), with the majority aged between 20 and 45 years (63.77%). Regarding socioeconomic status, 63.01% reported an average monthly income below 5,000 RMB, and 90.03% lacked any form of local basic medical insurance. Occupationally, participants were primarily concentrated in manufacturing (57.26%), followed by the construction and service industries (accounting for a combined 24.32%).

The serological testing group included 2,364 participants. The group was similarly predominantly male (58.97%), with 56.26% aged between 20 and 45 years. Although industry and income data were not collected for the serological survey, no statistically significant differences were observed between the two groups regarding key demographic variables such as age and place of household registration (*P* > 0.05), apart from a marginal difference in gender distribution (*P* = 0.047). These findings suggest that the questionnaire and serological testing samples were broadly comparable with respect to the available demographic characteristics.

### Descriptive results of questionnaire responses

Before evaluating the structural paths, we assessed the baseline descriptive distribution of the core constructs. As detailed in Table S1 in Supplementary Material S2, the surveyed migrant workers demonstrated varying levels of HBV knowledge, with only 36.7% and 31.2% fully identifying all transmission routes and preventive measures, respectively. A substantial proportion expressed willingness to undergo screening (91.6%) and treatment (98.1%). However, among those who reported being unwilling to utilize screening, vaccination, or clinical services, structural barriers were prominently cited. Time poverty emerged as the most frequently reported obstacle across all domains, particularly hindering screening (71.0%) and vaccination (52.7%), followed by economic constraints. Regarding other subjective psychosocial barriers assessed in the survey—such as fear of HBV detection, stigma, perceived unnecessity, and fear of job loss—these were rarely cited by participants, with positive response rates uniformly below 1.2%. Given this severe floor effect, these subjective indicators were excluded from subsequent structural modeling.

### Serological testing results and comparison with self-reported data

**Fig. 1:**
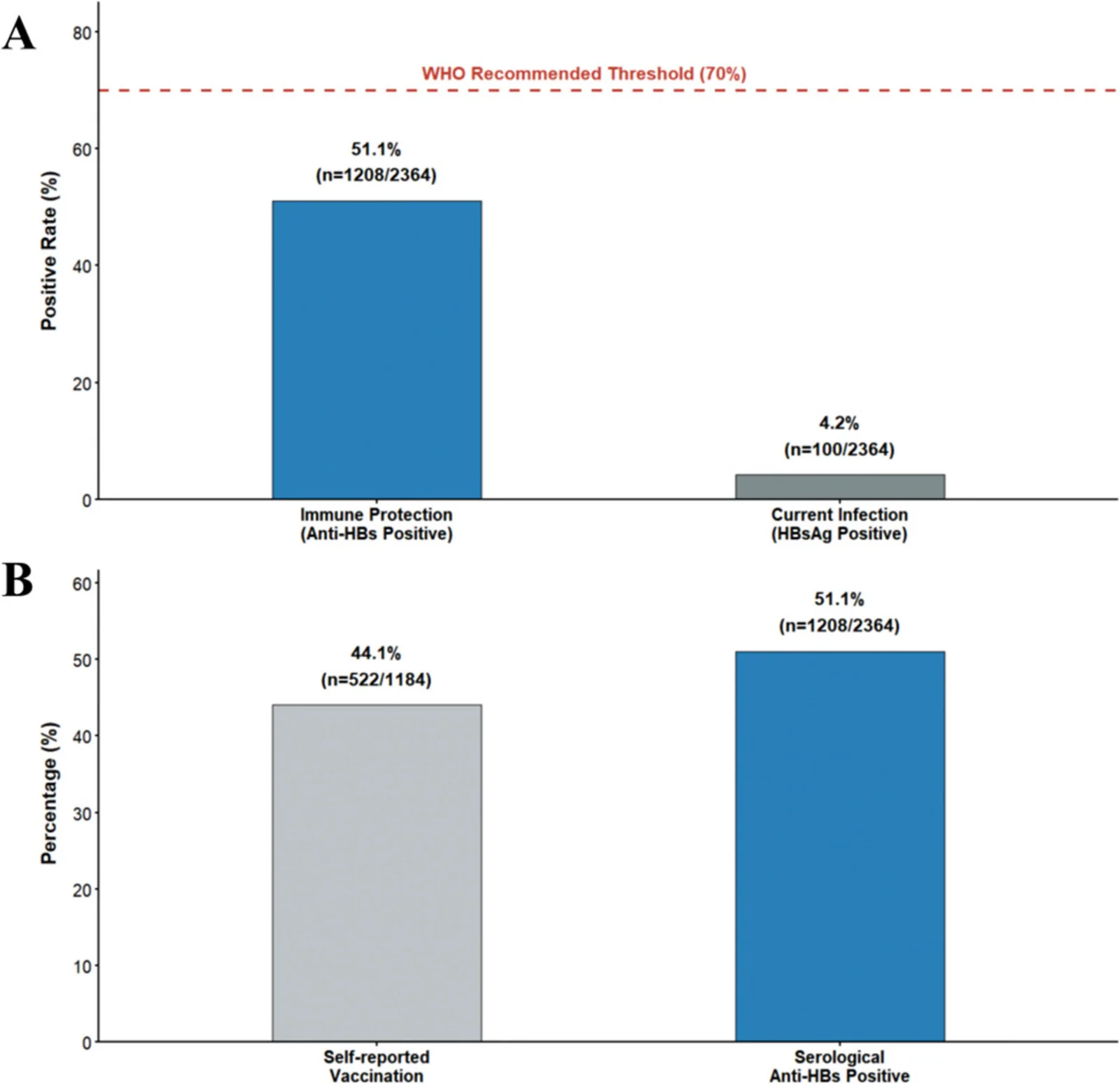
Serological markers of HBV and comparison with self-reported vaccination. (**A**) Prevalence of immune protection (anti-HBs positive) and current active infection (HBsAg positive) among migrant workers (N=2,364). The dashed line represents the 70% population immunity threshold recommended by the World Health Organization. (**B**) Comparison between self-reported vaccination history (N=1,184) and overall serologically confirmed anti-HBs positivity (N=2,364)

Among the 2,364 migrant workers tested, the positive rate for protective antibodies (anti-HBs) was 51.10% (1,208/2,364), which is substantially lower than the 70% population immunity threshold recommended by the World Health Organization (WHO) (Fig. 1A). Anti-HBs positivity differed significantly across age groups (χ² = 47.40, *P* < 0.001). The highest anti-HBs positive rate was observed among workers aged 26–35 years (62.1%, 360/580), followed by those aged 20–25 years (51.7%, 155/300) and 36–45 years (50.6%, 334/660). In contrast, lower levels of immune protection were observed among workers aged 46–55 years (42.3%, 199/470) and > 55 years (45.2%, 160/354). The positive rate for HBsAg, indicating current active infection, was 4.23% (100/2,364).

To evaluate the self-reported data in the context of actual biological protection, we compared the self-reported history of HBV vaccination with the overall serological anti-HBs positivity rate. The questionnaire survey revealed that 44.08% (522/1,184) of respondents self-reported having “previously received the hepatitis B vaccine.” This proportion was significantly lower than the actual serologically confirmed anti-HBs positive rate (51.10%) (χ^2^ = 15.34, *P* < 0.001) (Fig. 1B). Despite this statistical discrepancy, both metrics consistently indicated insufficient immune protection within this population.

### Spatial distribution of immune protection

**Fig. 2:**
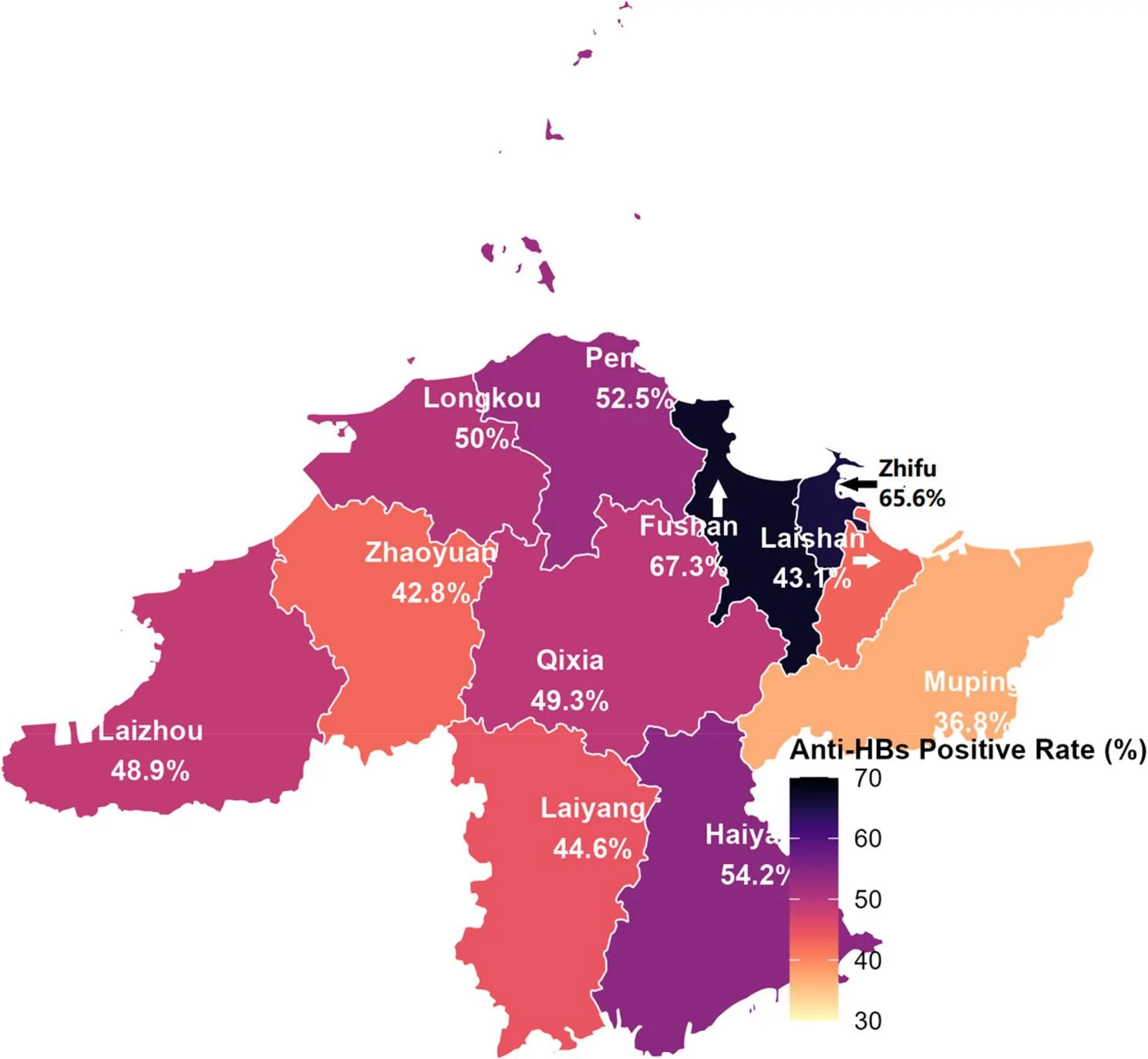
Spatial heatmap of anti-HBs positive rates across districts in Yantai City. Spatial heatmap illustrating the variation in regional anti-HBs positive rates across the 13 districts of Yantai City. Colors represent the gradient of seroprevalence, with darker shades indicating higher levels of immune protection

Analysis of anti-HBs positive rates in Yantai City revealed significant spatial heterogeneity (χ² = 74.38, *P* < 0.001), with a citywide average of 51.10% (1,208/2,364). As illustrated in Fig. 2, the highest seroprevalence was observed in the Fushan industrial belt north of the city center (67.33%, 101/150). Zhifu District, an established commercial center with dense community health infrastructure, reported the second-highest rate (65.6%). Conversely, the lowest prevalence was recorded in Muping District (36.81%, 53/144), a predominantly service-based area in the eastern urban region. Rates in other manufacturing-heavy districts, including Laizhou (48.8%) and Haiyang (54.2%), approximated the overall mean. Elevated immunity levels clustered in districts dominated by large-scale organized industries or comprehensive urban health networks, while lower rates were concentrated in areas reliant on dispersed, small-scale service enterprises (Table 2).

**Table 2:** Distribution of anti-HBs positive rates among migrant workers by district, Yantai City

District	No. tested	Anti-HBs positive *n* (%)	95% CI	Main industrial characteristics
Zhifu	305	200 (65.6)	60.2–70.9	Commerce, services
Fushan	150	101 (67.3)	59.8–74.8	Manufacturing (auto parts, equipment manufacturing)
Muping	144	53 (36.8)	28.9–44.7	Services (catering, tourism, retail)
Laishan	130	56 (43.1)	34.6–51.6	High-tech industries
High-tech Zone	30	11 (36.7)	19.4–53.9	High-tech industries
Longkou	240	120 (50.0)	43.7–56.3	Manufacturing, energy
Laiyang	260	116 (44.6)	38.6–50.7	Agriculture, food processing
Laizhou	260	127 (48.8)	42.8–54.9	Machinery manufacturing, stone materials
Penglai	120	63 (52.5)	43.6–61.4	Tourism, wine
Zhaoyuan	243	104 (42.8)	36.6–49.0	Gold industry
Qixia	140	69 (49.3)	41.0–57.6	Agriculture (apples)
Haiyang	192	104 (54.2)	47.1–61.2	Nuclear power, tourism
Huangbohai New Area	150	84 (56.0)	48.1–63.9	Manufacturing, high-tech
Total	2364	1208 (51.1%)	49.1–53.1	-

### Structural equation modeling analysis

Prior to evaluating the structural paths, CFA was conducted to assess the reliability and validity of the modified measurement model, which integrated objective HBV knowledge. The measurement model demonstrated excellent fit to the data (Robust CFI = 0.981, Robust TLI = 0.972, Robust RMSEA = 0.046, SRMR = 0.033). As presented in Table 3, all standardized factor loadings were statistically significant (P < 0.001), ranging from 0.532 to 0.841, indicating adequate indicator reliability. Internal consistency was acceptable, with Cronbach’ s α of 0.65 for Cues to Action, 0.79 for HBV Knowledge, and 0.82 for Preventive Intention. Furthermore, CR scores for Knowledge (0.80) and Intention (0.77) exceeded the recommended threshold of 0.70, and their AVE values were above 0.50 (0.68 and 0.53, respectively), establishing robust convergent validity for these core constructs. Although the ‘Cues to Action’ construct exhibited a slightly lower Cronbach’ s α (0.65) and AVE (0.32), this is theoretically acceptable, as disparate external information sources (e.g., internet versus family) function primarily as formative rather than highly correlated reflective indicators.

**Table 3:** Reliability and validity of the measurement model

Latent Constructs	Indicators	Standardized Factor Loadings	Cronbach’s α	Composite Reliability (CR)	Average Variance Extracted (AVE)
Cues to Action	Cue_Net	0.565^***^	0.65	0.66	0.32
	Cue_Comm	0.630^***^			
	Cue_Fam	0.543^***^			
	Cue_Media	0.532^***^			
HBV Knowledge	Know_Trans	0.822^***^	0.79	0.80	0.68
	Know_Prev	0.822^***^			
Preventive Intention	Will_Screen	0.744^***^	0.82	0.77	0.53
	Will_Vacc	0.841^***^			
	Will_Diag	0.627^***^			
	Will_Treat	0.594^***^			

^***^: *P* < 0.001

To test the underlying mechanisms driving hepatitis B protective behaviors and the effects of socioeconomic limitations, an MLR-based structural equation model (SEM) was estimated. To facilitate an assessment of model improvement, we first evaluated the fit indices for the zero-variable (null) baseline model. The null model yielded a poor fit to the data (Robust χ^2^ = 4161.135, df = 102, *P* < 0.001). In contrast, the adjusted structural model achieved a substantial reduction in the Chi-square statistic (Robust χ^2^ = 354.136, df = 76) and demonstrated an acceptable fit to the observed data: Robust CFI = 0.937, Robust TLI = 0.916, Robust RMSEA = 0.061 (90% CI: 0.055–0.067), and SRMR = 0.064, confirming the appropriateness of the structural framework.

**Fig. 3:**
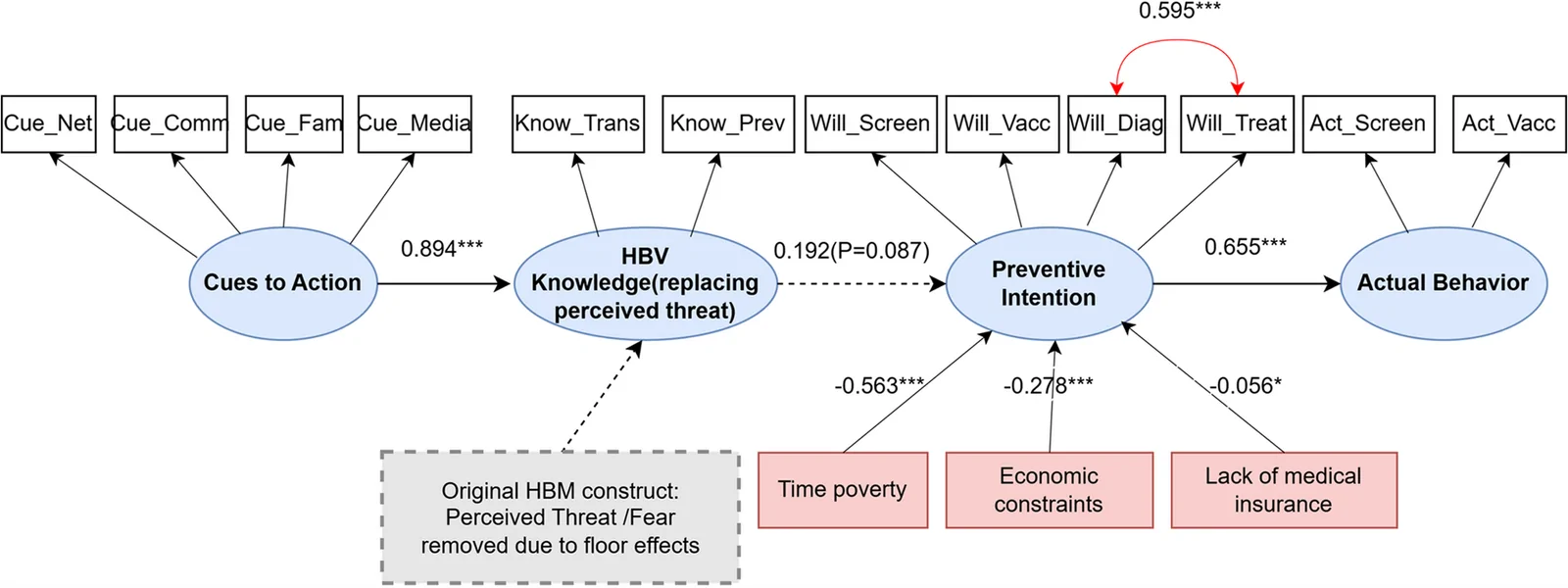
Path diagram of the structural equation model. Standardized path coefficients (β) are presented for the structural model. Solid lines indicate estimated paths, and the dashed line represents a non-significant path. Blue ovals denote latent constructs included in the final model, and pink rectangles denote observed exogenous covariates (structural barriers). The red double-headed arrow indicates the residual covariance between diagnostic and treatment intentions. The grey dashed box represents the original HBM perceived threat/fear construct, which was removed from the estimated model because of severe floor effects. HBV Knowledge was used as the replacement cognitive appraisal construct in the adapted model. Abbreviations: Cue_Net, internet/social media cues; Cue_Comm, community outreach/health campaign cues; Cue_Fam, family/friend cues; Cue_Media, television/radio cues; Know_Trans, knowledge of HBV transmission routes; Know_Prev, knowledge of HBV prevention measures; Will_Screen, willingness to undergo HBV screening; Will_Vacc, willingness to receive vaccination; Will_Diag, willingness to undergo diagnostic confirmation; Will_Treat, willingness to receive treatment; Act_Screen, self-reported HBV screening history; Act_Vacc, self-reported HBV vaccination history. *P < 0.05; ***P < 0.001

Standardized path coefficients (β) and their significance levels are presented in Table 4, and the corresponding path diagram is shown in Fig. 3. External Cues to Action were strongly and positively associated with HBV knowledge (β = 0.894, *P* < 0.001). However, HBV knowledge showed a positive but marginally non-significant association with preventive intention (β = 0.192, *P* = 0.087), suggesting that knowledge may contribute to preventive motivation but may not be sufficient on its own.

**Table 4:** Path coefficients of the structural equation model

Hypothesized path	Unstandardized coefficient (B)	S.E.	Z-value	Standardized coefficient (β)	*P*-value
HBV Knowledge ← Cues to Action	3.198	0.160	20.016	0.894	< 0.001
Preventive Intention ← HBV Knowledge	0.110	0.064	1.711	0.192	0.087
Preventive Intention ← Time poverty	-1.300	0.092	-14.190	-0.563	< 0.001
Preventive Intention ← Economic constraints	-0.841	0.101	-8.357	-0.278	< 0.001
Preventive Intention ← Lack of medical insurance	-0.106	0.049	-2.166	-0.056	0.030
Actual Behavior ← Preventive Intention	0.438	0.047	9.295	0.655	< 0.001

The modified model demonstrated acceptable fit to the data: Robust CFI = 0.937, Robust TLI = 0.916, Robust RMSEA = 0.061, and SRMR = 0.064

The model also provided quantitative evidence for the intention-action gap. Preventive intention significantly predicted actual behavior (β = 0.655, *P* < 0.001), confirming a functional pathway from motivation to action. Critically, however, this motivational process was substantially undermined at the intention-formation stage by objective structural barriers. Specifically, time poverty exerted the strongest negative effect on preventive intention (β = -0.563, *P* < 0.001), followed by economic constraints (β = -0.278, *P* < 0.001) and the lack of medical insurance (β = -0.056, *P* = 0.030).

## Discussion

By integrating self-reported data, serological biomarkers, and spatial analysis, this study shows that the gap between migrant workers’ hepatitis B prevention intentions and their actual behaviors is driven primarily by structural barriers. While objective knowledge remains a necessary foundation for health behavior, it is insufficient on its own to bridge this gap. The structural equation model confirmed that, when external constraints are absent, preventive intention does translate into actual behavior (β = 0.655). However, the formation of intention itself was substantially weakened by time poverty, economic constraints, and lack of medical insurance, with time scarcity exerting the strongest effect (β = −0.563). The serological and spatial evidence reinforced this picture: only 51.10% of workers had protective anti-HBs antibodies—well below the WHO-recommended 70% threshold [5, 18]—and immune protection varied across districts. Taken together, these findings indicate that this behavioral discrepancy is not explained by knowledge deficits alone, but is likely shaped by the interaction between cognitive motivation and socioeconomic constraints that keep willingness from turning into clinic visits.

Models such as the Health Belief Model assume that cognitive factors, including knowledge and perceived threat, can contribute to preventive motivation [7, 19]. In our data, HBV knowledge showed a positive but marginally non-significant association with preventive intention (β = 0.192, *P* = 0.087). Therefore, our findings suggested that knowledge alone may be insufficient to overcome structural constraints in this population. External cues to action were strongly linked to HBV knowledge acquisition (β = 0.894), indicating that health communication efforts may improve awareness. However, this increased awareness has not translated into correspondingly stronger health intentions. The pattern aligns with meta-analysis that changes in knowledge and attitudes are often insufficient to close the intention-behavior gap, especially in socioeconomically marginalized groups [8, 20]. The novel contribution of this study lies in its identification of specific structural factors that lead to a disconnect between knowledge and intention. The intentions reported by migrant workers do not stem from a lack of motivation or irrational health beliefs, but are constrained by the costs associated with taking action.

Among the three structural barriers, time poverty was the factor that most significantly influenced willingness to participate in prevention efforts, followed by financial constraints and lack of health insurance. This ranking reflects the realities of daily life for migrant workers in China. Most of them work in manufacturing, construction, or the service sector, where they face long working hours, strict shift schedules, and piece-rate pay [21]. Taking time off to visit a clinic for screening or vaccination often means losing income immediately, or even risking dismissal [22]. The fact that time outweighed cost does not mean cost is unimportant. It means that even when services are free—as the serological testing in this study was—the opportunity cost of time can still be too high for workers to afford [10]. This finding aligns with broader evidence that time is an underestimated social determinant of health, which can render nominally accessible services difficult to access in practice [23]. The effect of medical insurance was relatively small (β = −0.056). A likely explanation is that China’s basic health insurance schemes primarily cover inpatient care and offer limited reimbursement for outpatient preventive services [24]; consequently, having insurance has little effect on reducing perceived barriers to screening and vaccination [25].

Comparing self-reported vaccination history with actual serological anti-HBs test results provides an alternative perspective on this disconnect between motivation and behavior. It is crucial to note that anti-HBs positivity represents a composite of immunity acquired through either vaccination or past natural infection. Only 44.08% of questionnaire respondents said they had received the hepatitis B vaccine—significantly fewer than the 51.10% whose blood tests confirmed anti-HBs positivity. This discrepancy suggests that self-reporting, as the most common outcome measure in behavioral surveys, may underestimate the actual level of immune protection due to recall bias [11, 26]. Because our sequential survey and serological cohorts lacked individual-level linkage, we cannot definitively determine the extent to which this difference is driven by recall bias versus sampling variations between the two overlapping but non-identical groups. Therefore, the discrepancy between the self-reported vaccination rate (44.08%) and the actual immunity rate (51.10%) has two primary explanations. First, some workers may have received the vaccine through workplace vaccination campaigns or occupational health examinations but did not have it recorded in official vaccination records, leading to underreporting. Second, others likely acquired anti-HBs through natural infection without realizing they had been exposed. Regardless of the scenario, both point to the same conclusion: the level of immune protection among migrant workers in Yantai is clearly insufficient. This contrast underscores a practical point—objective biomarkers hold value in behavioral research, particularly among populations where recall bias and social expectations may distort respondent reports [27]. The age-stratified analysis further showed that immune protection varied across age groups. Anti-HBs positivity was highest among workers aged 26–35 years, whereas lower rates were observed among those older than 45 years. This pattern may reflect differences in vaccination opportunities across birth cohorts, as well as insufficient adult catch-up immunization. These findings suggest that older migrant workers should be considered a priority group for HBV screening and catch-up vaccination.

The spatial analysis further revealed that immune protection is not evenly distributed across different regions of Yantai but is closely linked to the local industrial structure [28]. Industrial agglomeration zones such as Fushan District, dominated by large-scale manufacturing, exhibited relatively high anti-HBs positivity rates (67.33%), whereas service-oriented districts such as Muping recorded much lower rates (36.81%). We hypothesize that this pattern could potentially reflect differential access to occupational health services [29]. Large manufacturing enterprises have standardized health management systems, pre-employment physical examinations, and routine vaccination programs covering all employees. In contrast, workers in service industries such as food service, retail, or tourism are more likely to be employed by small, scattered businesses, which are subject to weaker regulatory oversight and rarely implement organized health interventions. The pattern of immunity revealed by these data is not random. It highlights potential health inequalities influenced by the local economy. However, because variables measuring specific employer practices and workplace health services were not directly assessed in our survey, these contextual explanations remain plausible interpretations rather than established conclusions. These findings suggest that public health interventions for migrant workers need to be adapted to each district’s industrial mix and existing health infrastructure.

There are three implications for public health practice. First, drawn directly from our data, interventions aimed at closing this gap must take time poverty seriously. Offering free services is necessary but not enough; immunization and screening programs need to be delivered at times and places that fit workers’ schedules and locations. To achieve this, existing literature suggests that workplace-based models—on-site vaccination at factories and construction sites, mobile health units during shift breaks, extended hours at community health centers—can reduce the opportunity cost of taking part [30]. Embedding hepatitis B screening and vaccination into routine occupational health examinations, and requiring employers to provide paid time off for preventive care, would further lower the time barrier [31]. Second, the geographic variation in immune protection identified in our spatial analysis calls for spatially targeted resource allocation. Public health authorities can use spatial heatmaps to direct outreach to underserved districts, particularly those where employment is dominated by small, dispersed service-sector businesses. Third, the limited role of health insurance found in our model suggests that simply expanding coverage may not change preventive behavior much, unless reimbursement is extended to cover outpatient preventive services such as adult vaccination. Aligned with broader policy recommendations, reforming insurance schemes to reduce or eliminate out-of-pocket costs for hepatitis B screening and vaccination, alongside efforts to reduce time-related barriers, could together improve uptake [23, 24].

### Strengths and limitations

This study has several strengths. The triangulation design—combining survey, serological, and spatial data—allowed findings from different sources to complement each other at the population level. Serological biomarkers provided objective population-level evidence to complement self-reported behavior, and the spatial analysis uncovered structural inequalities that a questionnaire-based study would have missed. In addition, bringing quantifiable structural barriers directly into the SEM framework allowed us to move from describing the intention-action gap to explaining it.

Several limitations should also be acknowledged. First, the cross-sectional design precludes causal inference; the pathways identified in the SEM represent theoretically guided associations, not confirmed causal relationships. Second, because the questionnaire surveys preceded blood collection without retaining individual identifiers for anonymity, the two cohorts lacked exact individual-level matching. This precludes paired validation, meaning discrepancies between self-reported and serological data must be interpreted strictly as aggregate, ecological differences. Third, actual behavior was measured by self-reported screening and vaccination history, which is subject to recall bias. Fourth, the study was conducted in a single city in eastern China, which limits generalizability to migrant populations elsewhere. In addition, the study design may have introduced selection bias. Conducting the questionnaire before the blood collection could have inadvertently acted as a “cue to action,” artificially inflating subsequent testing participation. Additionally, the free serological testing likely attracted workers with existing health problems or greater health needs, while deterring individuals harboring HBV-related stigma or fear of occupational repercussions. Consequently, the observed seroprevalence may slightly deviate from the true parameters of the broader migrant worker population. This two-component design also required additional field coordination, personnel, laboratory testing, and communication with enterprises and workers. Fifth, the survey’s skip-logic restricted barrier assessment strictly to participants who expressed preventive unwillingness. Consequently, our model evaluated these constraints solely at the intention-formation stage, precluding analysis of their direct disruptive effects on actual behavior and likely underestimating their total impact across the care cascade.

## Conclusions

In conclusion, this study shows that the gap between migrant workers’ hepatitis B preventive intentions and their actual behavior is driven predominantly by structural barriers—above all, time poverty—that prevent intentions from turning into action. Reaching the WHO 2030 goal of eliminating viral hepatitis will require removing these barriers. That means delivering services at workplaces, directing resources to areas with the lowest coverage, and reforming insurance to reduce both the direct and indirect costs of prevention. For migrant workers facing high mobility, precarious employment, and limited access to care, closing the intention-action gap is not a matter of persuading individuals. It means changing the conditions in which they live and work.

## Supplementary Information

Supplementary Material 1.

Supplementary Material 2.

## Data Availability

The datasets used and/or analysed during the current study are available from the corresponding author on reasonable request.
