# Supplementary material for: Structural barriers drive the intention-action gap in hepatitis B prevention among migrants in Shandong Province, China: a triangulated study of serological, spatial, and behavioral data
Source: BMC Public Health. 2026 Jul 18;26:2957. doi: 10.1186/s12889-026-28627-z (PMC13663034; doi:10.1186/s12889-026-28627-z)
Supplement: Supplementary file 1 [file 12889_2026_28627_MOESM1_ESM.docx]

Supplementary Material S2: Descriptive results of of HBV knowledge, preventive intentions, and associated barriers.

Table S1. Descriptive statistics of HBV knowledge, preventive intentions, and associated barriers (N = 1,184)

| Variable | Response Category | N | Proportion (%) |
| --- | --- | --- | --- |
| **HBV Knowledge** |  |  |  |
| Knowledge of transmission routes | Full knowledge (Score = 4) | 434 | 36.7 |
|  | Incomplete / Unknown (Score < 4) | 750 | 63.3 |
| Knowledge of preventive measures | Full knowledge (Score = 4) | 370 | 31.2 |
|  | Incomplete / Unknown (Score < 4) | 814 | 68.8 |
| **Screening** |  |  |  |
| Willingness to undergo screening | Willing | 1084 | 91.6 |
|  | Unwilling | 100 | 8.4 |
| Primary barriers to screening* | Time poverty | 71 | 71.0 |
|  | Economic constraints | 29 | 29.0 |
|  | Lack of medical insurance | 13 | 13.0 |
| **Vaccination** |  |  |  |
| Willingness to receive vaccination | Willing | 1110 | 93.8 |
|  | Unwilling | 74 | 6.2 |
| Primary barriers to vaccination* | Time poverty | 39 | 52.7 |
|  | Economic constraints | 23 | 31.1 |
|  | Lack of medical insurance | 7 | 9.5 |
| **Diagnosis** |  |  |  |
| Willingness to seek diagnosis | Willing | 1155 | 97.6 |
|  | Unwilling | 29 | 2.4 |
| Primary barriers to diagnosis* | Time poverty | 16 | 55.2 |
|  | Economic constraints | 12 | 41.4 |
|  | Lack of medical insurance | 0 | 0.0 |
| **Treatment** |  |  |  |
| Willingness to receive treatment | Willing | 1161 | 98.1 |
|  | Unwilling | 23 | 1.9 |
| Primary barriers to treatment* | Time poverty | 11 | 47.8 |
|  | Economic constraints | 5 | 21.7 |
|  | Lack of medical insurance | 1 | 4.3 |

*Note: Barrier proportions are calculated exclusively among participants who reported being "Unwilling" to utilize the respective service (Scores 1-2 on a 4-point Likert scale). The proportions for barriers may exceed 100% cumulatively as multiple reasons could be selected.
