# Supplementary material for: Structural barriers drive the intention-action gap in hepatitis B prevention among migrants in Shandong Province, China: a triangulated study of serological, spatial, and behavioral data
Source: BMC Public Health. 2026 Jul 18;26:2957. doi: 10.1186/s12889-026-28627-z (PMC13663034; doi:10.1186/s12889-026-28627-z)
Supplement: Supplementary file 2 [file 12889_2026_28627_MOESM2_ESM.docx]

Supplementary Material S1: Questionnaire for HBV Prevention, Screening, Diagnosis, and Treatment Willingness among Migrant Workers in Yantai, China

Dear Participants:

Hello! We are conducting this survey to understand the willingness of internal migrant workers to participate in hepatitis B virus (HBV) prevention, screening, diagnosis, and treatment services. This survey is anonymous, and your responses will be used strictly for academic research. Please answer according to your actual situation. Thank you for your time and support!

**I. Basic Information**

1.Gender:

A. Male B. Female

2.Age:

A. 20–25 years B. 26–35 years C. 36–45 years D. 46–55 years E. Over 55 years

3.Province of Origin (Hometown): ____________________

4.Length of Employment in Yantai:

A. Less than 1 year B. 1–3 years C. 3–5 years D. More than 5 years

5.Planned Duration of Stay in Yantai:

A. Less than 1 year B. 1–3 years C. 3–5 years D. More than 5 years

6.Current Industry Sector:

A. Manufacturing B. Construction C. Service Industry (e.g., catering, domestic services) D. Logistics & Transportation E. Others

7.Monthly Average Income (RMB):

A. Below 3,000 B. 3,000–5,000 C. 5,001–10,000 D. Over 10,000

8.Are you currently living with chronic Hepatitis B?

A. Yes B. No C. Uncertain

9.Do you have local basic medical insurance in Yantai?

A. Yes B. No

**II. HBV Knowledge and Behavior History**

1.Have you ever heard of Hepatitis B?

A. Yes B. No

2. Do you know the possible transmission routes of hepatitis B virus (HBV)? (Select all that apply)

A. Blood transmission, such as sharing needles or receiving blood transfusions

B. Mother-to-child transmission

C. Sexual transmission

D. Daily casual contact, such as sharing tableware or hugging

E. Not sure

3. Which measures do you think can prevent hepatitis B virus (HBV) infection? (Select all that apply)

A. Receiving the hepatitis B vaccine

B. Avoiding unsafe sexual behavior

C. Avoiding unnecessary injections, blood transfusions, or other medical procedures D. Maintaining personal hygiene, such as frequent handwashing

E. Not sure

4.Have you ever undergone an HBV screening (blood test for HBsAg)?

A. Yes B. No C. Uncertain

5.Have you ever received the Hepatitis B vaccine?

A. Yes (Total ___ doses) B. No C. Uncertain

6.What are the main sources of your HBV-related information? (Select all that apply) A. Television/Radio B. Internet/Social Media C. Community Outreach/Health Campaigns D. Family/Friends

**III. HBV Screening Willingness and Barriers**

1.If provided with an opportunity, are you willing to undergo an HBV screening test (blood test for HBsAg)?

A. Very willing (Skip to Section IV)

B. Somewhat willing (Skip to Section IV)

C. Somewhat unwilling

D. Very unwilling

2.If you are unwilling, what are the primary reasons? (Select all that apply)

A. Inability to afford the screening costs (Economic constraint)

B. Lack of time/Work schedule conflicts (Time poverty)

C. Fear of being detected with HBV (Psychological barrier)

D. Fear of social stigma or discrimination (Psychological barrier)

E. Belief that no symptoms mean no need for screening (Cognitive barrier)

F. Others: ____________________

**IV. HBV Vaccination Willingness and Barriers**

1.If you are tested and found to have no HBV infection and no protective antibodies, are you willing to receive the Hepatitis B vaccine?

A. Very willing (Skip to Q3)

B. Somewhat willing (Skip to Q3)

C. Somewhat unwilling

D. Very unwilling

2.If you are unwilling, what are the primary reasons? (Select all that apply)

A. Concerns about vaccine safety

B. Perception of low infection risk/unnecessary

C. Inability to afford the high vaccination costs

D. Lack of time/Work schedule conflicts

E. Fear of social stigma or discrimination

F. Others: ____________________

3.Do you hope your family members will receive the Hepatitis B vaccine?

A. Very much B. Somewhat C. Not really D. Not at all

4.Which vaccination service mode do you prefer?

A. On-site collective vaccination at the workplace

B. Designated clinics/hospitals

C. Community clinics near the current residence

D. Returning to hometown for vaccination

**V. HBV Diagnosis and Treatment Willingness**

1.If a screening result suggests a potential HBV infection, are you willing to undergo further clinical diagnostic confirmation?

A. Very willing (Skip to Q3)

B. Somewhat willing (Skip to Q3)

C. Somewhat unwilling

D. Very unwilling

2.If you are unwilling, what are the primary reasons? (Select all that apply)

A. Inability to afford the high diagnostic costs

B. Lack of time for clinical visits

C. Fear of being detected with HBV

D. Fear of losing current employment if infected

E. Belief that no symptoms mean no need for diagnosis

3.If diagnosed with chronic Hepatitis B, are you willing to receive standardized medical treatment?

A. Very willing

B. Somewhat willing

C. Somewhat unwilling

D. Very unwilling

4.If you are unwilling to receive treatment, what is the main reason?

A. Inability to afford the high treatment costs

B. Lack of insurance coverage for outpatients

C. Concerns about side effects of medications

D. Others: ____________________
